# Prescriptions

**Published:** 1896-01

**Authors:** 


ALBUMINURIA (BRIGHT'S DISEASE).

℞. Ferri sulph.............gr. xv.

Magn. sulph.............℥ij.

Potass. bicarb...........ʒiij.

Infusi buchu.............℥viij.

M. Sig.:—A tablespoonful once or twice a day in a tumblerful of water. (*When constipation exists.*) —FOTHERGILL.

ANÆMIA AND CHLOROSIS.

℞. Tinct. ferri mur..........ʒiv.

Acidi phosphor. dil.......ʒvj.

Spts. limonis.............ʒij.

Syrupi............q. s. ad ℥vj.

M. Sig.:—A dessert spoonful in water after meals.—GOODELL.

CATARRH, NASAL AND FAUCIAL.

℞. Acidi carbolici liq.....Mxxx.

Sodii biborat..........

Sodii bicarb..........aa ʒj.

Glycerinæ............ʒiiiss.

Aquæ........q. s. ad ft. ℥iv.

M. Sig.:—To be used with atomizer. (*Simple chronic rhinitis.*)—DOBELL.

FROSTBITE.

℞. Acidi carbolici...........ʒj.

Tinct. iodinii.............ʒij.

Acidi tannici.............ʒij.

Cerati simplicis..........℥iv.

M. Ft. ungt. Sig.:—Apply locally. —BARTHOLOW.

---

See the Special Club

Rates offered cash subscribers, and our great premium offer on insert.

#### LEUCORRHŒA.

℞. Zinci sulphatis............ʒ iss.

Aluminis sulphatis.......ʒ iss.

Glycerinæ................℥ vj.

M. Sig.:—A tablespoonful to a quart of water, as a vaginal injection.—T. G. THOMAS.

#### LARYNGISMUS STRIDULUS.

℞. Potassii citratis........ʒ j.

Syr. ipecac.............ʒ ij.

Tinct. opii deod........gtt. xij.

Syr. simplicis..........ʒ ij.

Aquæ.................℥ iss.

M. Sig.:—A teaspoonful every two hours at two years of age. (*In severe form.*)—MEIGS AND PEPPER.

#### ENLARGED LYMPHATIC, GLANDS.

℞. Potassii iodidi..........ʒ i–iv.
Syr. aurantii cort........℥ j.
Aquæ cinnamomi....ad ℥ iij.
M. Sig.:—A teaspoonful in water three times daily.—RINGER.

#### LUMBAGO.

℞. Olei terebinthinæ......ʒ ii–iij.

Mucilag. acaciæ, q.s. ut.ft.emuls

Syr. zingiberis.........℥ j.

Aquæ.................ad ℥ iij.

M. Sig.:—A tablespoonful every four to six hours, carefully, lest strangury and nephritis supervene. (*When urine is clear and abundant, and bowels regular.*)—WARING.

